# Laser-scanning cytometry can quantify human adipocyte browning and proves effectiveness of irisin

**DOI:** 10.1038/srep12540

**Published:** 2015-07-27

**Authors:** Endre Kristóf, Quang-Minh Doan-Xuan, Péter Bai, Zsolt Bacso, László Fésüs

**Affiliations:** 1 MTA-DE Stem Cells, Apoptosis and Genomics Research Group of the Hungarian Academy of Sciences, Department of Biochemistry and Molecular Biology, University of Debrecen, Debrecen, Hungary; 2 Department of Biophysics and Cell Biology, University of Debrecen, Debrecen, Hungary; 3 MTA-DE Lendület Laboratory of Cellular Metabolism Research Group, Research Center for Molecular Medicine, Department of Medical Chemistry, University of Debrecen, Debrecen, Hungary

## Abstract

Laser-scanning cytometry is presented as a tool allowing population scale analysis of *ex vivo* human brown adipogenic differentiation. It combines texture analysis and detection of Ucp1 protein content in single brown adipocytes of mixed cell populations with gene expression pattern and functional characteristics of browning. Using this method we could validate mouse data in human samples demonstrating the effectiveness of irisin to induce “beige” differentiation of subcutaneous white adipocytes.

The importance of brown adipose tissue (BAT) in controlling the energy homeostasis of mice and also the adult human body has become increasingly evident in the past few years, highlighting the possibility of therapeutic application of BAT stimulation in the treatment of obesity and diabetes mellitus^1^. Recent studies focused on BAT differentiation in mouse models found that there are at least two different types of thermogenic adipocytes, “classical brown” and “beige”, which have different developmental origins^2^. In line with this, deep neck BAT in the adult human shares many similarities with murine “classical” BAT, while other active, heat producing fat depots have the molecular characteristics of murine “beige” cells^3^,^4^. Although, a protocol to induce human brown adipocyte differentiation in cell culture was developed several years ago^5^, there is only limited data about regulatory networks that drive, or mediators that influence human “classical brown” or “beige” adipocyte differentiation^6^,^7^. Using cellular, in particular *ex vivo* human models, and performing population scale analysis to determine the heterogeneity of cultured and differentiated primary adipocytes in response to certain stimuli would be a valuable tool to understand the development of “classical brown” or “beige” adipocytes in distinct human adipose tissue depots and to validate novel findings obtained in mice.

Several investigators have successfully used flow cytometry to assess surface protein expression of human primary adipocytes or to separate floating adipocytes from stromal-vascular cells^8^,^9^. In spite of its analytical power, no concise protocol is available to identify brown adipocytes in a large population of cells by flow cytometry due to the fact that widely accepted surface markers have not been described, and the collection of precise morphological data (e.g. evaluation of the size or number of lipid droplets in single adipocytes) and the possibility to inspect adipocyte differentiation at several time points are sacrificed under the experimental conditions it requires. Furthermore, already attached adipocytes are too fragile to tolerate detachment by trypsinization^10^. In order to overcome these limitations, we aimed to quantify brown adipocyte differentiation *ex vivo* at a single cell level in a highly replicative manner by using a slide-based image cytometry approach.

Human adipose-derived mesenchymal stem cells (hADMSCs) were cultivated from abdominal subcutaneous fat^11^. Highly confluent hADMSCs were treated in chambered slides with optimized white^12^ or brown^5^ adipogenic differentiation cocktails. As a result, up to 50% of the treated hADMSCs progressively accumulated lipid droplets. To analyze the samples at consecutive time points we turned to laser-scanning cytometry (LSC)^13^ which combines scanning lasers, a microscope and automated image acquisition and inherits both the cytometric attributes of flow cytometry and the photographing operation of a microscope^14^,^15^,^16^. This technique is thus not limited to analyzing cells in flowing fluids^17^ and allows automated analysis of large population of cells with minimized perturbation. LSC can perform high-content analysis of adherent cells cultured in a chambered slide^18^,^19^. From captured images of the samples, the fluorescent and/or chromatic signals can be evaluated through a series of segmentation algorithms. Cellular objects are recognized and handled along with their measured cytometric parameters, including morphological properties, such as object size, shape, location and texture as well as intensity profiles. In our case, first fluorescently labelled nuclei of cells were identified, then cellular morphology, lipid accumulation and major brown adipogenic marker protein expression were inspected simultaneously, as shown in Fig. 1a and Supplementary Fig. S1. Immunofluorescent staining showed that Ucp1 was distributed in punctate structures between the lipid droplets of differentiated brown adipocytes, while Cidea accumulated highly in the perinuclear lipid-free region of these cells. Moreover, characteristic texture properties of differentiated adipocytes were analyzed. Parameter variance measures the difference between the signal intensity of a central pixel and its neighborhood; hence “sum variance” reflects the size of lipid droplets^13^ as illustrated in Supplementary Fig. S2. We found significantly lower texture “sum variance” along with the accumulation of smaller lipid droplets as a result of *ex vivo* brown adipocyte differentiation. There was no significant difference between white and brown adipocytes in respect of the overall size of the cells. The brown differentiation cocktail lead to a two-fold higher Ucp1 and Cidea protein content in single human adipocytes compared to white differentiation (Fig. 1b). In Fig. 1c, we plotted Ucp1 or Cidea immunofluorescence intensity and texture “sum variance” for each differentiated adipocyte. Brown adipocytes were identified as the ones that contained small lipid droplets and high levels of Ucp1 or Cidea protein (lower right quadrant of density plot images), in contrast to white adipocytes, which accumulated large lipid droplets and expressed low amount of brown adipogenic marker proteins (upper left quadrant of density plot images). Using this approach, we demonstrated that depending on individual donors 30–70% of the human primary adipocytes that accumulated lipid droplets gained the characteristics of brown cells as a result of *ex vivo* brown adipogenic differentiation. The biological variance among donors of hADMSCs was described in Supplementary Table S1. In parallel, we selected to analyze the expression of a core set of brown fat-specific genes (*Ucp1*^20^*, Cidea*^21^*, Pgc1a*^22^, *Elovl3*^23^) and a marker of mitochondrial enrichment (*Cyc1*^20^). The expression of *Prdm16*^24^ and *Cebpb*^25^ which are transcriptional regulators of brown adipocyte development was also assessed. A “beige” marker (*Tbx1*^4^) and a “classical brown” adipocyte marker gene (*Zic1*^3^) were investigated as well. Finally, key drivers of the white and the general adipogenic program (*Cebpa*^26^*, Pparg*^27^) and white (*Lep*) or general (*Fabp4*) adipogenic markers^28^ were examined. Upregulation of several major brown adipocyte marker genes (*Ucp1, Cidea, Cyc1, Elovl3, Pgc1a*) (Fig. 1d, Supplementary Fig. S3, Supplementary Table S2), 5-fold higher expression of Ucp1 protein (Fig. 1e; n = 3, p < 0.05, calculated by densitometry of immunoblots) and significantly higher mitochondrial DNA amount (Fig. 1f) were found in whole cell lysates of *ex vivo* differentiated human brown adipocytes. In addition, we found induction of *Tbx1*, a “beige” marker gene^4^, while the expression of *Zic1*, a “classical brown” adipocyte marker gene^3^ was not altered as a result of *ex vivo* brown adipogenic differentiation (Supplementary Fig. S3, Supplementary Table S2). The significant upregulation of *Tbx1* suggests that human primary adipocytes from abdominal subcutaneous fat follow the „beige” pathway when differentiated according to the protocol developed by Elabd *et al.*^5^.

Using this method, we intended to clarify the direct effect of irisin and BMP7 on the induction of browning in human adipocytes. Irisin was discovered as a myokine which is cleaved from the Fndc5 transmembrane protein and induces a browning program in subcutaneous white adipose tissue in mice^29^. The human *Fndc5* gene carries a G to A mutation in the ATG start codon. This conversion probably results in a shorter Fndc5 protein lacking the part from which irisin is generated^6^. However, several studies have demonstrated the presence of irisin in human blood plasma, using many different antibodies, as a function of exercise or other metabolic parameters^30^,^31^,^32^. Besides that, inconsistent effects were found when recombinant Fndc5 or irsin was administered directly to differentiating human adipocytes^6^,^7^,^33^. BMP7 was described earlier as a locally acting mediator in mice that drives “classical brown” adipogenesis and recruits “beige” adipocytes^34^. In our experiments, both irisin and BMP7 treatment during white adipocyte differentiation significantly upregulated *Ucp1*, *Elovl3*, *Cidea*, *Cyc1* and *Pgc1a* genes (Fig. 2a,b, Supplementary Fig. S4). Furthermore, elevated expression of *Zic1*, *Cebpb*, *Prdm16*, *Cebpa*, and *Pparg* genes was found in whole cell lysates of BMP7 treated adipocytes (Fig. 2c, Supplementary Figs S4-S5). Expression of *Tbx1* increased selectively as a result of irisin administration (Fig. 2d). In line with this, irisin treated differentiating white adipocytes contained smaller lipid droplets and higher amount of Ucp1 and Cidea protein than the untreated cells (Fig. 3a,b). Similar changes were found as a consequence of BMP7 administration except for the decrease in texture “sum variance” which did not exceed the level of statistical significance (Fig. 3b). Next, we analyzed Ucp1 immunofluorescence intensity and texture “sum variance” of 2000 differentiated adipocytes in 3 different donors as depicted on Fig. 3c. 30–60% of these cells had the characteristic morphological features of *ex vivo* differentiated brown adipocytes as a result of irisin or BMP7 treatment (Supplementary Table S1). Our results suggest that irisin is able to induce a “beige” program in differentiating human primary subcutaneous white adipocytes, while BMP7 induces a “classical brown” adipocyte-like phenotype. Finally, we intended to investigate the functional capacity of human primary adipocytes differentiated in the presence of irisin, BMP7 or brown adipogenic differentiation protocol as described above. Irisin or BMP7 treated differentiating white adipocytes also had increased mitochondrial DNA, to a similar extent as brown adipocytes (Supplementary Fig. S6). These cells had significantly increased cAMP stimulated mitochondrial respiration compared to white adipocytes. Interestingly, basal mitochondrial respiration of irisin treated white adipocytes and *ex vivo* differentiated brown adipocytes was already elevated as compared to the untreated white cells (Fig. 3d, Supplementary Fig. S7).

To date, white, “classical brown” or “beige” adipocyte development has mostly been evaluated based on the detection of mRNA or protein expression in whole cell lysates. However, up to 50% of precursor cells remain undifferentiated in human cellular models of white or brown adipocyte development. Furthermore, our results clearly demonstrate that the population of *ex vivo* differentiated adipocytes remains heterogeneous regardless of whether white or brown adipogenic differentiation was induced. Taking this fact into consideration, as far as we are aware, our LSC based method is the first approach that can clearly discriminate between human white and brown adipocytes in cell culture conditions in a high throughput manner. By complementing measurements of gene expression changes from total cell lysates, our LSC technique helps to specifically identify brown cells in order to quantify human brown adipocyte differentiation effectively in a population scale manner and validate data that were obtained by using mouse models. Here, we used this approach to demonstrate that irisin is able to induce a “beige” phenotype (characterized by high content of small lipid droplets and brown adipogenic marker proteins) in human white adipocytes. In the future this method can help researchers to sort out homogenously differentiated hADMSC populations not only providing the possibility to understand the differences between “classical brown” or “beige” differentiation pathways in humans but also to “engineer” thermogenically active, transplantable brown adipocytes to aid weight reduction in obese individuals.

## Methods

### Materials

All chemicals were from Sigma-Aldrich (Munich, Germany) unless stated otherwise.

### Ethics Statement

Human adipose-derived mesenchymal stem cells (hADMSCs) were isolated from subcutaneous abdominal adipose tissue of healthy volunteers (body mass index <29.9) aged 20–65 years who underwent a planned surgical treatment (herniotomy). Written informed consent from all participants was obtained before the surgical procedure. The study protocol was approved by the Ethics Committee of the University of Debrecen, Hungary (No. 3186-2010/DEOEC RKEB/IKEB). All experiments were carried out in accordance with the approved ethical guidelines and regulations.

### Isolation of hADMSCs, Cell Culture and *ex vivo* Differentiation Induction

Subcutaneous abdominal adipose tissue samples were immediately transported to the laboratory following herniotomy. Adipose tissue specimens were dissected from fibrous material and blood vessels, minced into small pieces and digested in PBS with 120 U/ml collagenase for 60 min in a 37 °C water bath with gentle agitation. The completely disaggregated tissue was filtered (pore size 140 μm) to remove any remaining tissue. The cell suspension was centrifuged for 10 min at 1300 rpm, and the pellet of stromal cells (hADMSCs) was resuspended in DMEM-F12 medium containing 10% FBS (Gibco), 100 U/ml penicillin-streptomycin, 33 μM biotin and 17 μM panthothenic acid. hADMSCs were seeded into 6-well plates or Ibidi eight-well μ-slides (Ibidi GmbH, Planegg/Martinsried, Germany) at a density of 15000 cells/cm^2^ and cultured in the same medium at 37 °C in 5% CO_2_ for 24 h to attach. Floating cells were washed away with PBS and the remaining cells were cultured until they became confluent^11^,^13^. The absence of mycoplasma was checked by polymerase chain reaction (PCR) analysis (PCR Mycoplasma Test Kit I/C, PromoKine, PromoCell France). White adipocyte differentiation was induced for four days using the following medium: DMEM-F12 supplemented with 33 μM biotin, 17 μM panthothenic acid, 10 μg/ml human apo-transferrin, 20 nM human insulin, 100 nM cortisol, 200 pM triiodothyronine, 2 μM rosiglitazone (Cayman Chemicals, Ann Arbor, MI, USA), 25 nM dexamethasone and 500 μM 3-isobutyl-1-methylxanthine. After four days rosiglitazone, dexamethasone and 3-isobutyl-1-methylxanthine were omitted from the differentiation medium^11^,^12^,^13^. Brown adipogenic differentiation was carried out for three days using the following medium: DMEM-F12 containing 33 μM biotin, 17 μM panthothenic acid, 10 μg/ml apo-transferrin, 0.85 μM human insulin, 200 pM triiodothyronine, 1 μM dexamethasone and 500 μM 3-isobutyl-1-methylxanthine. Three days later, the medium was changed (dexamethasone and 3-isobutyl-1-methylxanthine were omitted) and 500 nM rosiglitazone was added^5^. From this point on media were changed every other day and cells were used after 14 days of differentiation. As a next step, white or brown adipocytes were differentiated in the presence of 250 ng/ml human recombinant irisin (Cayman Chemicals) or 50 ng/ml human recombinant BMP7 (R&D systems, Minneapolis, USA)^6^,^29^. Irisin or BMP7 was administered during the whole differentiation procedure, or in the last 4 days of the differentiation.

### Immunofluorescence Staining

hADMSCs were plated on Ibidi eight-well μ-slides and differentiated as previously described. On the day of measurement, cells were washed once with phosphate buffered saline (PBS) and then kept in fresh medium for sub vital scanning with 50 μg/ml Hoechst 33342 for 60 minutes and 750 μg/ml Nile Blue for 20 minutes. Next, cells were washed and fixed in 4% paraformaldehyde for 5 min followed by staining with anti-Ucp1 or anti-Cidea (Covalab, Villeurbanne, France) primary antibodies for 6 h at room temperature. Alexa 488 goat anti-rabbit IgG (Invirogen Life Technologies) was used as a secondary Ab. Antibodies were applied and additional washing steps between and after Ab usage were performed in the presence of 0.1% saponin in PBS for effective cell permeabilization.

### Image Acquisition

Images were obtained by using iCys Research Imaging Cytometer (iCys, Thorlabs Imaging Systems, Sterling, VA, USA). Sample slides were mounted on a computer-controlled stepper-motor driven stage. An area with optimal confluence was determined in low-resolution scout scan with a × 10 magnification objective (NA 0.30) and a 10-μm scanning step. High-resolution images were consequently obtained by using a × 40 objective (NA 0.75) and a 0.25-μm scanning step. The size of a pixel was set at 0.25 μm × 0.245 μm at × 40 magnification.

Laser lines were separately operated, namely a 405-nm diode laser was used to excite Hoechst 33342, a solid-state 488-nm laser was used for Alexa 488 goat anti-rabbit IgG and a 633-nm HeNe gas laser for Nile Blue. Emissions were collected by three photomultiplier tubes; Hoechst was detected at 450 ± 20 nm, Alexa 488 at 530 ± 15 nm and Nile Blue at above 650 nm. Transmitted laser light was captured by diode photodetectors in which light loss and shaded relief signals were measured to gain information about light absorption, light scattering and texture of the objects^13^.

### Automated Recognition of Cellular Objects

Images were processed and analyzed by our high throughput automatic cell recognition protocol using the iCys companion software (iNovator Application Development Toolkit, CompuCyte Corporation, Westwood, MA) and CellProfiler (The Broad Institute of MIT, MA).

Hoechst-stained nuclei were first identified and marked as primary objects. Based on parent nuclei, the secondary objects, a whole cell, were then recognized according to its Nile Blue fluorescence^13^ (Supplementary Fig. S1).

### Texture Analysis, Assessment of Ucp1 and Cidea Protein Content of Single Adipocytes, Quantification of *ex vivo* Brown Adipocyte Differentiation

2000–3000 cells per sample were collected for image analysis. Clustered, detached or dead cells were omitted and 1000–2000 cells were quantified per data set. Textural pattern of light absorption and light scatter signal per identified objects was analyzed with built-in modules in CellProfiler. Parameter variance measured the difference between intensity of the central pixel and its neighborhood; “sum variance” (ΣV)^35^ roughly depicted the size of lipid droplets^13^ as illustrated in Supplementary Fig. S2. Beside texture parameters, the other major profiles that were also extracted are: Integral, which is the sum of the pixel intensities for a given event that provides information about the expression level of the labelled protein; and Area, which is the area enclosed by the boundary contour of the object, in square micrometers^36^. From these parameters, Ucp1 or Cidea immunofluorescence intensity per cell could be assessed as the value of the Integrated intensity relative to the Area of each event. Ucp1 or Cidea intensities and texture “sum variance” of each differentiated adipocyte were then plotted. Cells were identified as brown adipocytes if their texture “sum variance” was lower than 3–4.5 and their Ucp1 immunofluorescence intensity was higher than 7500–9000 (in arbitrary units and visualized in the lower right quadrant of density plot images).

### RNA Preparation and TaqMan Real-time RT-PCR

Total cellular RNA was isolated from hADMSCs and differentiated white or brown adipocytes using TRIzol Reagent (Invitrogen Life Technologies). Total RNA concentrations were quantified by spectrometry after DNase treatment. TaqMan reverse transcription reagent kit (Applied Biosystems) was used for generating cDNA according to manufacturer’s instructions. An ABI Prism 7700 sequence detection system (Applied Biosystems) was used to determine relative gene expression of “classical brown”, “beige”, white and general adipocyte markers. Gene primers and probes were designed and supplied by Applied Biosystems. Human *Gapdh* was used as endogenous control. All samples were run in triplicate. Gene expression values were calculated based on the ΔΔCt method, where one sample was designated as calibrator, through which all other samples were analyzed. ΔCt represents the threshold cycle (Ct) of the target minus that of *Gapdh* and ΔΔCt represents the ΔCt of each target minus that of the calibrator. Relative quantities were determined using the equation where relative quantity equals 2^−ΔΔCt^.

### Immunoblotting

hADMSCs and differentiated adipocytes were washed with PBS and collected followed by lysing in 50 mMTris–HCl; 0.1% Triton X-100; 1 mM EDTA; 15 mM 2-MEA and proteinase inhibitors. Insoluble cellular material was removed by centrifugation and the lysates were mixed with 5 × Laemmli loading buffer (LB), boiled for 10 min and loaded onto a 10% SDS polyacrylamide gel. Proteins were transferred onto PVDF membranes followed by blocking with 5% skimmed milk. Membranes were probed by polyclonal anti-Ucp1 and monoclonal anti-GAPDH (Millipore, Billerica, MA, USA) antibodies overnight at 4 °C, followed by incubation with horseradish-peroxidase (HRP)-conjugated species-corresponding secondary antibodies for 1 h at room temperature. Immunoblots were developed with Immobilon Western chemiluminescent substrate (Millipore). Densitometry was carried out using Image J software.

### mtDNA Quantification by Quantitative Real-Time PCR

Total cellular DNA was isolated by a conventional phenol-chloroform method from hADMSCs and differentiated adipocytes using TRIzol Reagent. Quantitative PCR was performed in triplicate on diluted DNA using 10 μM each primer (mtDNA specific PCR, forward 5′-CTATGTCGCAGTATCTGTCTTTG-3′, reverse 5′-GTTATGATGTCTGTGTGGAAAG-3′; and nuclear specific PCR (*Sirt1* gene), forward 5′- CTTTGTGTGCTATAGATGATATGGTAAATTG-3′, reverse 5′- GATTAAACAGTGTACAAAAGTAG-3′) and Maxima SYBR Green/ROX qPCR Master Mix (Thermo Scientific) in a LightCycler 480 (Roche Diagnostics, Mannheim, Germany) with a program of 20 minutes at 95 °C, followed by 50 cycles of 15 seconds at 95 °C, 20 second at 58 °C and 20 second at 72 °C. Single-product amplification was verified by an integrated post-run melting curve analysis. Results were calculated from the difference in threshold cycle (CT) values for mtDNA and nuclear specific amplification. Data are expressed as mitochondrial genomes per diploid nuclei^37^.

### Oxygen Consumption

Oxygen consumption was measured using an XF96 oximeter (Seahorse Biosciences, North Billerica, MA, USA). hADMSCs were seeded and differentiated in 96-well XF96 assay plates. On the day of measurement, after recording the baseline oxygen consumption, cells received a single bolus dose of dibutyril-cAMP (500 μM final concentration) simulating adrenergic stimulation. Then, stimulated oxygen consumption was recorded every 30 minutes. The final reading took place at 7 h post-treatment. As a last step, cells received a single bolus dose of Antimycin A (10 μM final concentration) for baseline correction. The oxygen consumption rate was normalized to protein content and normalized readings were displayed^37^.

### Statistical analysis

Results are expressed as the mean ± SD for the number of assays indicated. For multiple comparisons of groups statistical significance was calculated and evaluated by one-way ANOVA followed by Tukey post-hoc test. In comparison of two groups Student’s t-test was used.

## Additional Information

**How to cite this article**: Kristóf, E. *et al.* Laser-scanning cytometry can quantify human adipocyte browning and proves effectiveness of irisin. *Sci. Rep.* **5**, 12540; doi: 10.1038/srep12540 (2015).

## Supplementary Material

Supplementary Information

## Figures and Tables

**Figure 1:**
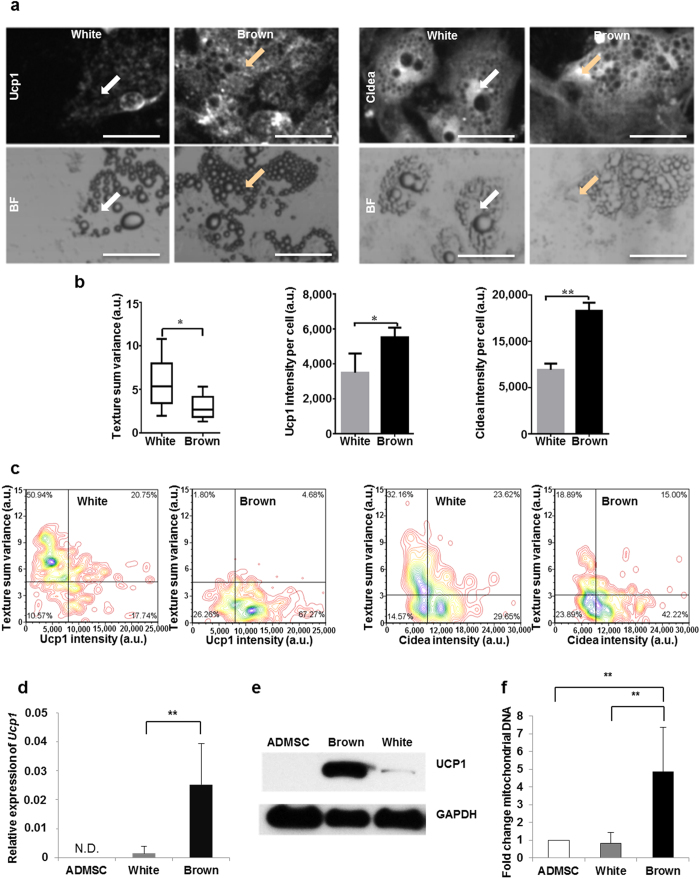
Laser-scanning cytometry based population scale analysis of *ex vivo* human brown adipocyte differentiation **(a)** Distribution of lipid droplets and Ucp1 or Cidea in differentiated adipocytes. Brown cells (representative examples are indicated with brown arrows) accumulated smaller lipid droplets and more Ucp1 or Cidea protein then the white adipocytes (representative examples are indicated with white arrows). Bars represent 50 μm. **(b)** Texture “sum variance”, Ucp1 and Cidea protein content of adipocytes per cell. n = 6, 1000–2000 cells per each sample **(c)** Dot plot figures of one representative donor based on which brown adipocytes can be identified as cells containing small lipid droplets and high levels of Ucp1 or Cidea. **(d)** *Ucp1* gene expression in human adipocytes. n = 10 **(e)** Ucp1 protein expression in one representative adipocyte donor. **(f)** Relative mitochondrial DNA amount of human adipocytes (as compared to undifferentiated hADMSCs). n = 7; Results are expressed as the mean ± SD for the number of assays indicated. For comparison of two groups statistical significance was evaluated by Student’s t-test. *p < 0.05, **p < 0.01.

**Figure 2:**
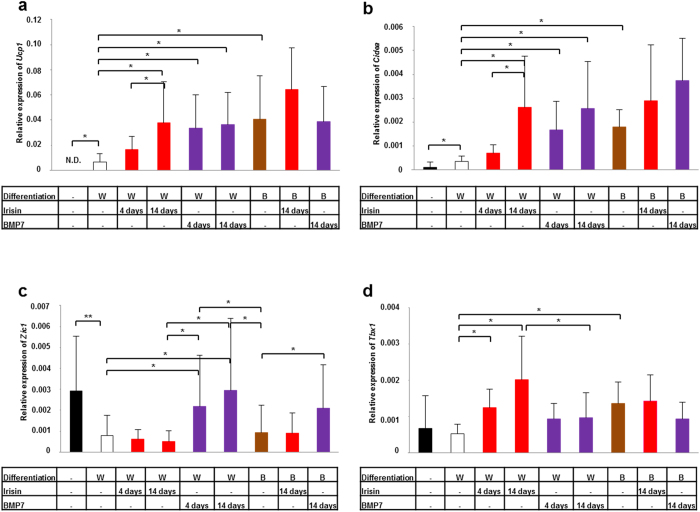
Expression of *Ucp1* (a), *Cidea* (b), *Zic1* (c) and *Tbx1* (d) in *ex vivo* differentiated human adipocytes treated with irisin or BMP7. ADMSCs were differentiated for two weeks to white (W) or brown (B) adipocytes. 250 ng/ml irisin (red bars) or 50 ng/ml BMP7 (blue bars) was administered on the last 4 days or during the whole differentiation process. Target genes were normalized to GAPDH. n = 5; Results are expressed as the mean ± SD for the number of assays indicated. For multiple comparisons of groups statistical significance was evaluated by one-way ANOVA followed by Tukey post-hoc test. *p < 0.05, **p < 0.01.

**Figure 3:**
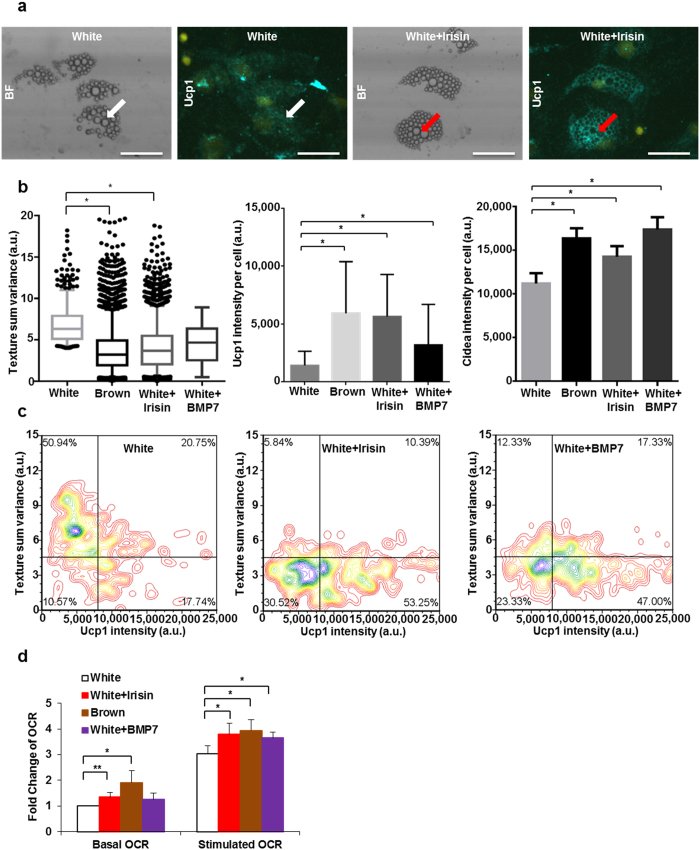
Population scale analysis of *ex vivo* differentiated primary adipocytes treated with irisin or BMP7. **(a)** Distribution of lipid droplets and Ucp1 in irisin treated white adipocytes. Irisin treated cells (a representative example is indicated with a red arrow) accumulated smaller lipid droplets and more Ucp1 protein then the untreated white adipocytes (a representative example is indicated with a white arrow). Bars represent 50 μm. **(b)** Texture “sum variance”, Ucp1 and Cidea protein content of adipocytes per cell. n = 3, 1000–2000 cells per each sample **(c)** Dot plot figures showing texture sum variance and Ucp1 content of differentiated cells in one representative donor. Brown adipocytes were identified as in Fig. 1c. (**d**) Basal and stimulated oxygen consumption level (as compared to basal OCR of white adipocytes) of adipocytes. n = 3; Results are expressed as the mean ± SD for the number of assays indicated. For multiple comparisons of groups statistical significance was evaluated by one-way ANOVA followed by Tukey post-hoc test. *p < 0.05, **p < 0.01.
