# Supplementary material for: Laser-scanning cytometry can quantify human adipocyte browning and proves effectiveness of irisin
Source: Sci Rep. 2015 Jul 27;5:12540. doi: 10.1038/srep12540 (PMC4515591; doi:10.1038/srep12540)
Supplement: Supplementary Information [file srep12540-s1.pdf]

Supplementary Information

Laser-scanning cytometry can quantify human adipocyte browning and proves effectiveness of irisin

Endre Kristóf, Quang-Minh Doan-Xuan, Péter Bai, Zsolt Bacso & László Fésüs*

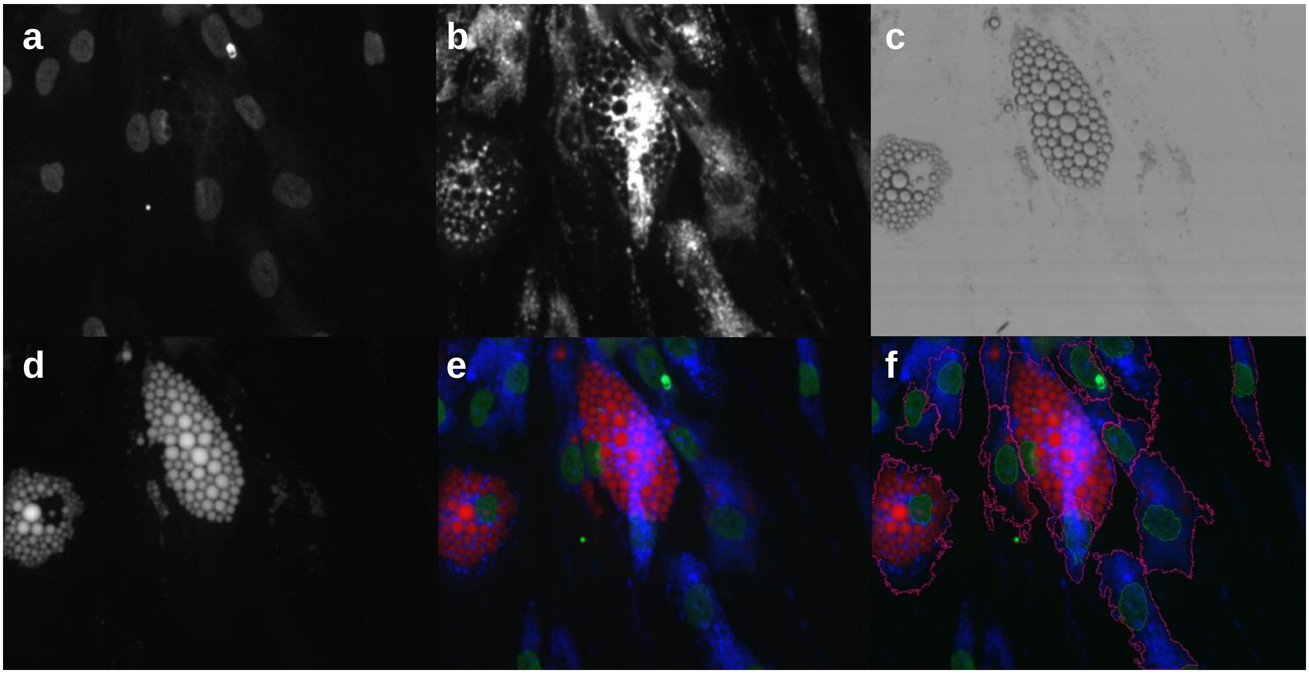

Supplementary Figure 1 Segmentation and automated recognition of cellular objects in adipocytes by laser-scanning cytometry. (a) Hoechst 33342-stained nuclei of differentiated adipocytes and preadipocytes. **(b)** Phospholipid specific Nile Blue labelling. **(c)** Transmitted light images by which texture analyses were performed. **(d)** Nile Red staining as an alternative approach to visualize and analyze lipid droplets. **(e)** Merged image of Hoechst, Nile Blue and Nile Red channels. **(f)** Nuclei were identified as primary objects, contoured with green lines. Then, secondary objects as adipocytes and preadipocytes were detected using Nile Blue signal in close association with the predefined primary objects and bordered with red lines. Cell recognition was performed by the automated segmentation module of CellProfiler software.

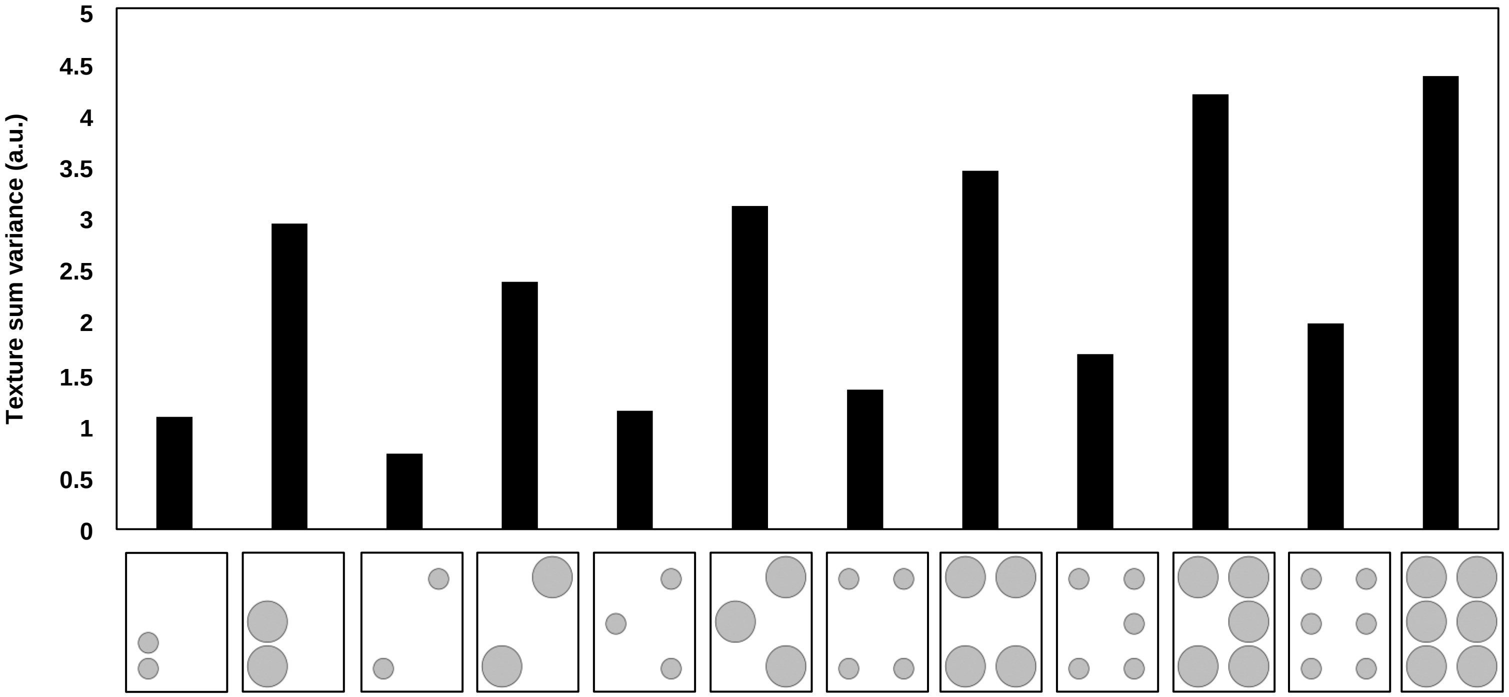

Supplementary Figure 2 Illustration of how texture sum variance reflects the size of lipid droplets in a cell-by-cell analysis. 12 rectangular white objects (representing cells) are shown which have the same size (10x7.5 inch). The droplets are simulated by grey dots in 2 fixed sizes: either 1.5 inch or 3.0 inch diameter. Each virtual cell contains exclusively 1 type of droplet size. The texture analysis of these cells (texture scale probes were set at 0.8 inch) shows that higher values of texture sum variance correspond to larger droplets. The effect of distance between the droplets is also demonstrated: cells that contain scarce distribution of droplets have lower values of texture sum variance compared to cells that contain clustered droplets.

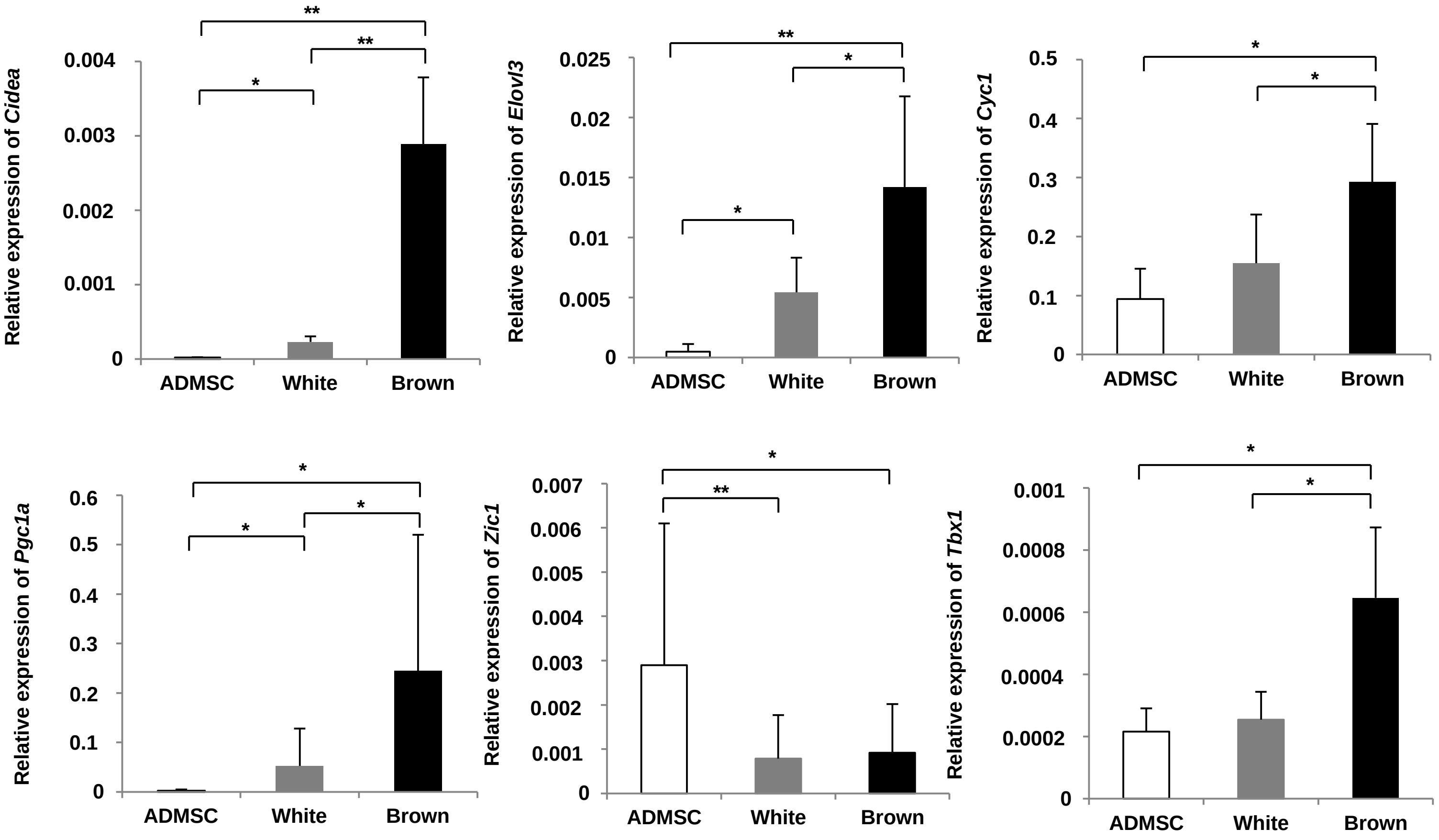

Supplementary Figure 3 Changes in expression levels of brown and beige adipogenic marker genes in primary human adipocytes during *ex vivo* brown adipocyte differentiation. ADMSCs of 10 different donors were differentiated for two weeks to white or brown adipocytes. (Target genes were normalized to GAPDH) *p<0.05, **p<0.01

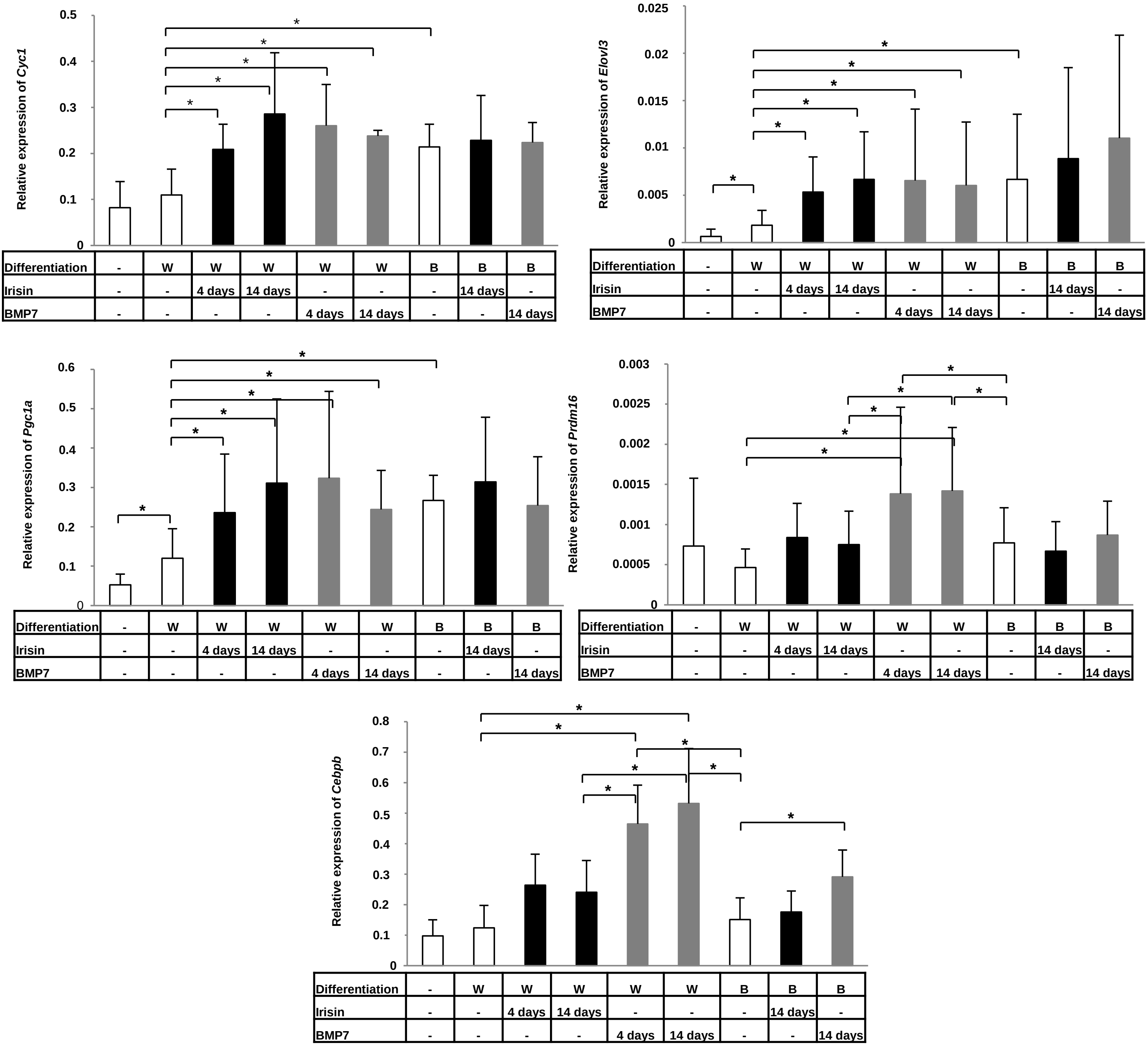

Supplementary Figure 4 Relative expression of brown adipogenic markers and key transcriptional regulators of brown adipocyte development in primary human adipocytes as a result of irisin or BMP7 treatment during *ex vivo* white (W) or brown (B) adipocyte differentiation. 250 ng/ml irisin (black bars) or 50 ng/ml BMP7 (grey bars) was administered on the last 4 days or during the whole differentiation process. (Target genes were normalized to GAPDH) *$p<0.05$, $n=5$.

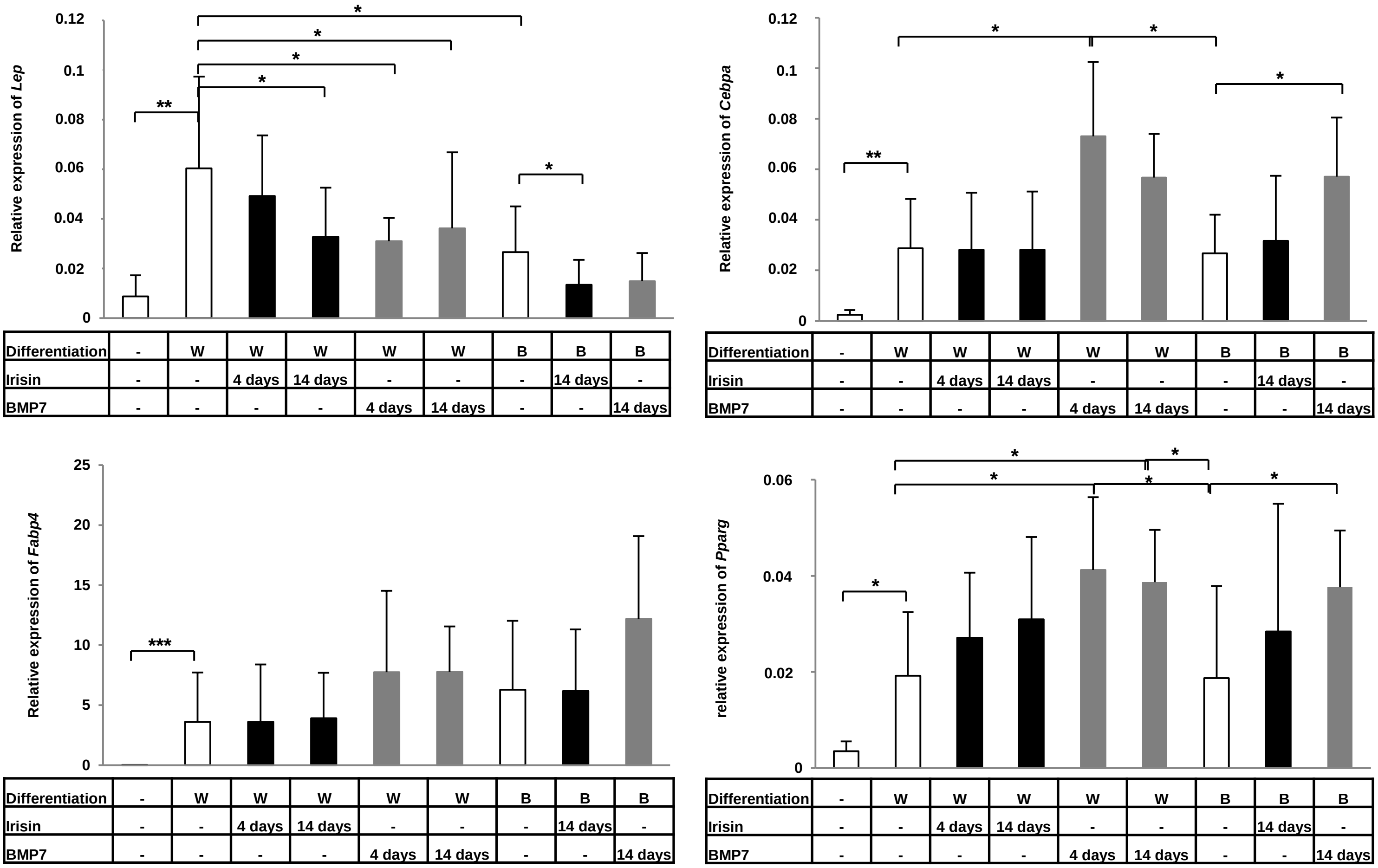

Supplementary Figure 5 Relative gene expression of white and general adipogenic markers in primary human adipocytes as a result of irisin or BMP7 treatment during *ex vivo* white (W) or brown (B) adipocyte differentiation. 250 ng/ml irisin (black bars) or 50 ng/ml BMP7 (grey bars) was administered on the last 4 days or during the whole differentiation process. (Target genes were normalized to GAPDH) *p<0.05, **p<0.01, ***p<0.001, n=5.

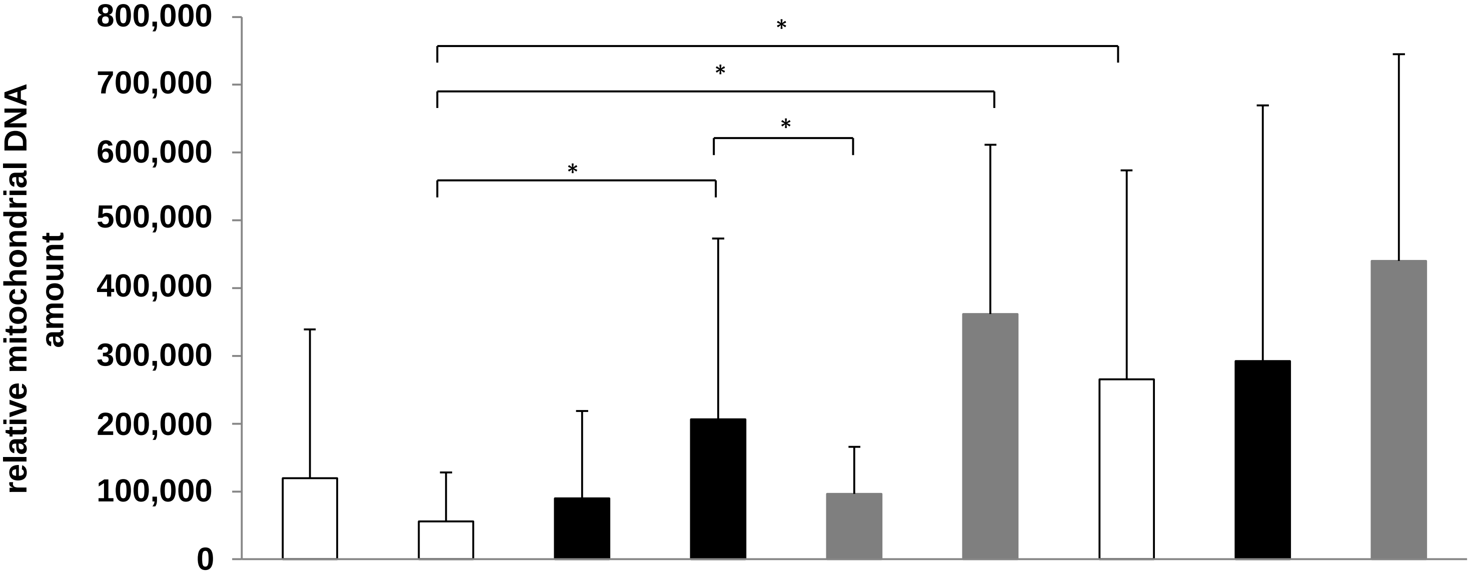

Differentiation	-	W	W	W	W	W	B	B	B
Irisin	-	-	4 days	14 days	-	-	-	14 days	-
BMP7	-	-	-	-	4 days	14 days	-	-	14 days

Supplementary Figure 6 Relative mitochondrial DNA amount of *ex vivo* differentiated primary human adipocytes determined by qPCR. ADMSCs were differentiated for two weeks to white (W) or brown (B) adipocytes. 250 ng/ml irisin (black bars) or 50 ng/ml BMP7 (grey bars) was administered on the last 4 days or during the whole differentiation process. *$p<0.05$, n=5

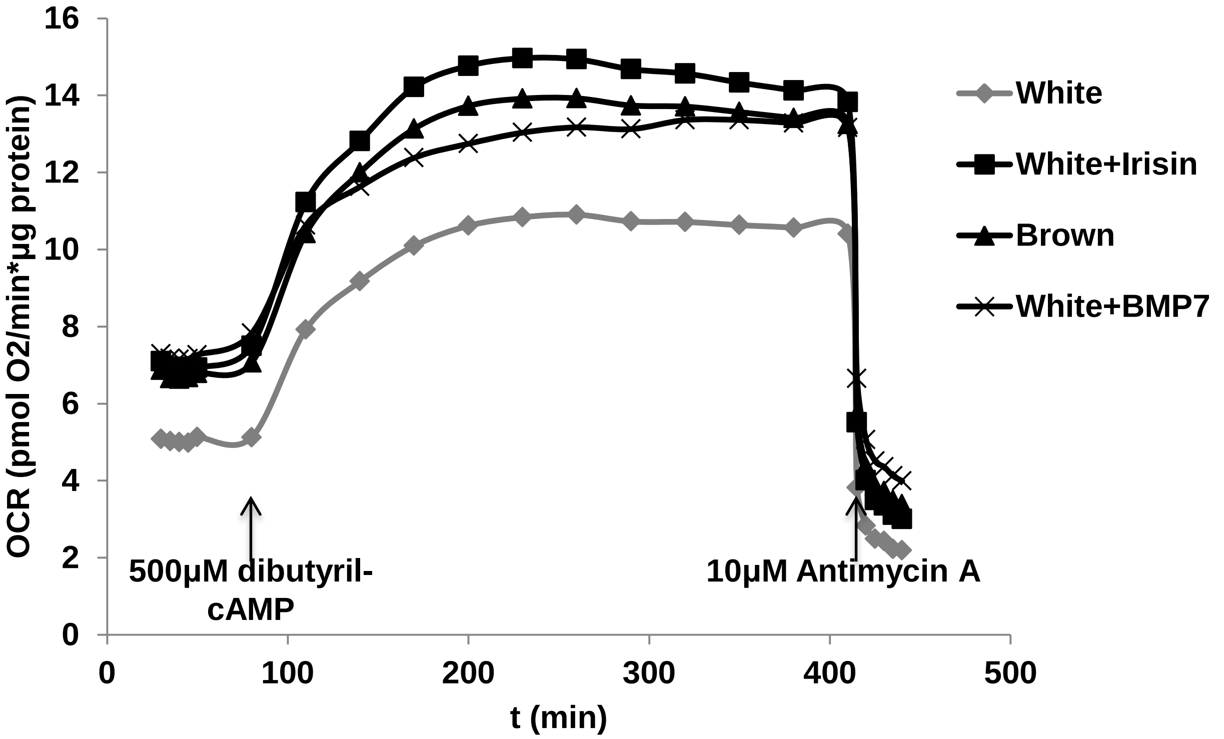

Supplementary Figure 7 Functional analysis of *ex vivo* differentiated primary adipocytes treated with irisin or BMP7. Oxygen consumption of one representative ADMSC derived adipocyte donor measured by an XF96 oximeter. After recording the baseline oxygen consumption, cells received a single bolus dose of dibutyril-cAMP (500 µM final concentration) modelling adrenergic stimulation. Then, stimulated oxygen consumption was recorded every 30 min. The oxygen consumption rate was normalized to protein content and normalized readings were displayed.

Donors	White differentiation protocol		Brown differentiation protocol		White differentiation protocol + Irisin		White differentiation protocol + BMP7	
	% of differentiated adipocytes							
	Texture↑ Ucp1↓	Texture↓ Ucp1↑	Texture↑ Ucp1↓	Texture↓ Ucp1↑	Texture↑ Ucp1↓	Texture↓ Ucp1↑	Texture↑ Ucp1↓	Texture↓ Ucp1↑
**1**	43.89	10.56	33.89	24.44	16.68	42.10	12.17	26.97
**2**	50.94	17.74	1.80	67.27	5.84	53.25	12.33	47.00
**3**	36.54	9.62	6.78	55.93	10.53	37.78	8.86	31.58

Supplementary Table 1 Laser Scanning Cytometry based population scale analysis of ex vivo adipogenic differentiation by texture parameters and Ucp1 protein content of primary adipocytes showing the biological variance of different donors. Brown adipocytes are identified as they contain small lipid droplets (Texture↓) and high level of Ucp1 protein (Ucp1↑).

GENES	ADMSC Average	SD	White Average	SD	FC (W/ADMSC)	Brown Average	SD	FC (B/ADMSC)	FC (B/W)
Ucp1	N.D.	-	0.0036	0.0048	N.D.	0.026	0.014	N.D.	7.26 **
Elovl3	0.00051	0.00018	0.0055	0.0027	10.71 *	0.014	0.0062	27.95 **	2.61 *
Cyc1	0.094	0.041	0.15	0.059	1.65	0.29	0.087	3.12 *	1.89 *
Cidea	0.000018	0.0000076	0.00023	0.000082	12.72 *	0.0029	0.00071	159.13 **	12.51 **
Pgc1a	0.0023	0.0011	0.054	0.072	23.85 *	0.25	0.34	111.86 **	4.69 *
Prdm16	0.00046	0.00065	0.00072	0.00023	1.58	0.00077	0.00038	1.69	1.07
Cebpb	0.092	0.044	0.12	0.056	1.47	0.15	0.062	1.96	1.33
Zic1	0.0029	0.0032	0.00079	0.00098	0.27**	0.00092	0.0011	0.32 *	1.16
Tbx1	0.00022	0.000069	0.00025	0.000093	1.18	0.00065	0.00018	2.99 *	2.54 *
Lep	0.0087	0.0042	0.061	0.038	7.02 **	0.026	0.016	2.99 *	0.43 *
Cebpa	0.0023	0.00078	0.029	0.017	12.61 **	0.027	0.015	11.74 **	0.93
Fabp4	0.00073	0.00034	3.61	2.84	4945.52 ***	6.27	4.67	8589.56 ***	1.72
Pparg	0.0018	0.00073	0.0096	0.0081	4.51 *	0.0094	0.011	5.27 *	0.98

Supplementary Table 2 Numerical data of changes in expression levels of adipogenic genes in primary human adipocytes during *ex vivo* white or brown adipocyte differentiation. Table shows average relative expression levels of ADMSCs, white and brown adipocytes, their standard deviation (SD) and fold changes (FC) as a ratio of expression levels of white adipocytes and ADMSCs (W/ADMSC); brown adipocytes and ADMSCs (B/ADMSC); brown adipocytes and white adipocytes (B/W). *$p<0.05$, **$p<0.01$, *** $p<0.001$, n=10. (Target genes were normalized to GAPDH)
